# Statistical density modification with non-crystallographic symmetry

**DOI:** 10.1107/S0907444902016360

**Published:** 2002-11-26

**Authors:** Thomas C. Terwilliger

**Affiliations:** a Mail Stop M888, Los Alamos National Laboratory, Los Alamos, NM 87545, USA

**Keywords:** density modification, non-crystallographic symmetry

## Abstract

Statistical density modification is a technique for phase improvement through a calculation of the posterior probability of the phases, given experimental phase information and expectations about features of the electron-density map. The technique can take advantage of both estimates of electron density in the map and uncertainties or probability distributions for those estimates. For crystals with non-crystallographic symmetry (NCS), this allows the use of the expected similarity of electron density at NCS-related points without requiring an implicit assumption that these regions are identical.

## Abstract 2

Statistical density modification can make use of NCS in a crystal and can include estimates of the deviations from perfect NCS.

## 1. Introduction

Non-crystallographic symmetry (NCS) can be a powerful aid in improving the quality of macromolecular electron-density maps (Bricogne, 1974 ▶; Rossmann, 1972 ▶; Kleywegt & Read, 1997 ▶). When present, NCS is often used along with solvent flattening (Wang, 1985 ▶) as a constraint on the electron density in a map, resulting indirectly in an improvement of the phases. Largely because it is difficult to do otherwise, in this process the NCS is generally treated as if it were exact, even if it might not be or if NCS-related density might be more similar in one region than in another. In the holographic method of Szöke *et al.* (1997 ▶), the possibility of imperfect NCS was introduced into the density-modification process by using a cost function to describe the expected similarity of NCS copies. In the method of Abrahams & Leslie (1996 ▶), imperfect NCS was also considered in weighting the various NCS copies during averaging.

Recently, we developed a method for improving crystallographic phases through the use of expectations about the electron density in a map that can take advantage of both the estimates of electron density in the map and uncertainties or probability distributions for those estimates (Terwilliger, 1999 ▶, 2000 ▶). This ‘statistical density-modification’ technique (previously known as ‘maximum-likelihood density modification’) combines experimental phase probabilities with phase probabilities derived from the expectations about the electron-density map to yield posterior (combined) phase probabilities. The key elements in this method are the use of a map-probability function to describe the plausibility of an electron-density map and the calculation of derivatives of the probability function to describe how this plausibility would change if an individual phase were changed.

The map-probability function in statistical density modification consists of the integral over all points in the map of a local log probability of the map. In turn, the local log probability of the map is the logarithm of the *a priori* probability of the value of the electron-density map at that point. The *a priori* probability distributions for plausible values of the electron density at each point in the map can come from any source, including the flatness of the solvent, the expected range of electron densities in the region of a macromolecule and, in the present case, non-crystallographic symmetry. As the map-probability function uses probability distributions rather than expected electron densities, it takes into account both the expected electron density (the mean of the probability distribution) and uncertainties in this expectation (through the distribution itself). In the case of NCS, this means that statistical density modification can take NCS into account without requiring an implicit assumption that the NCS is exact or even that the deviations from perfect NCS are the same everywhere in the region where NCS applies.

## 2. Methods

### 2.1. Identification of NCS operators.

Non-crystallographic symmetry operators were identified using the NCS in heavy-atom sites (Terwilliger, 2002 ▶). Additionally, approximate centers of regions where they would apply were estimated from the centroids of the coordinates of each of the *n*_NCS_ sets of heavy-atom sites that could be related to another set of heavy-atom sites through NCS. These NCS operations were then checked by determining the covariance of density in regions related by the NCS operators as a function of distance *d* from the (approximate) centers of the regions where NCS applies, fitting this covariance to a Gaussian centered at *d* = 0, extrapolating the covariance to *d* = 0 and only including NCS if the extrapolated covariance 〈ρ_*i*_ρ_*j*_〉 was at least 0.1 times the mean-square value of the electron density in the map (it was typically 0.5 to 2 times the mean-square value of the map).

### 2.2. Identification of ‘NCS asymmetric unit’

The region over which NCS applies and which is repeated *n*_NCS_ times in the asymmetric unit of the crystal was identified using the covariance in a fashion similar to that used for checking the NCS operators and similar to the automatic method described by Cowtan (1998 ▶). A local mean covariance of density among the *n*_NCS_ regions of NCS was used to identify this region. For each point on a grid centered at the center of one of the presumed regions where NCS applies, the mean value of the covariance of density 〈ρ_*i*_ρ_*j*_〉 for a sphere with radius of *r* around all pairs of points related by NCS to this one was calculated. The radius *r* was typically taken to be the same as the radius used for smoothing the squared electron density in mask calculation for solvent flattening (Wang, 1985 ▶). The NCS asymmetric unit was then defined by sequentially testing all points on the grid, starting with those close to the origin and then moving further away. If the point had a mean covariance of density greater than a cutoff *c*_MIN_ and was not related by crystallographic symmetry or NCS to any other point already in the NCS asymmetric unit, it was included. The cutoff *c*_MIN_ was chosen by testing a range of values and picking the one that yielded approximately the same fraction of the unit cell within the *n*_NCS_ regions of NCS as was expected to be within the macromolecule (*i.e.* not solvent) region of the unit cell.

### 2.3. Estimation of expected electron-density probability distribution from NCS

The electron density ρ_*i*_ at a point *i* related by NCS symmetry to *n*_NCS_ − 1 other points in the asymmetric unit was estimated from the weighted mean of the density at the *n*_NCS_ − 1 NCS-related points. The weights and the uncertainty in this estimate were estimated from the local covariance of density 〈ρ_*i*_ρ_*j*_〉 mentioned above as follows. A simple error model for the relationship between the density ρ_*i*_ and ρ_*j*_ at two NCS-related points *i* and *j* was used,
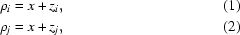
where *x* corresponds to the contribution to electron density that is shared by NCS-related points and *z*_*i*_ and *z*_*j*_ correspond the contributions that are unique to NCS copies *i* and *j* (including both errors in the map and true differences). The expected value of *x* ^2^ can then be estimated from the covariance of ρ_*i*_ and ρ_*j*_,
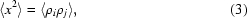
where the average is taken over all points in the NCS asymmetric unit and all pairs of NCS copies *i* and *j*. The expected value of 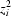 can then be estimated using (1), again averaged over the NCS asymmetric unit,
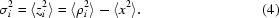
Using this error model, if ρ_*i*_ is used as an estimate of the electron density shared by all NCS copies for this point (*x*), then the expected variance in this estimate of *x* is given by 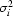.

This variance was used as the weighting term for averaging the *n*_NCS_ − 1 values of electron density used to estimate the value of *x* for each point in the NCS asymmetric unit and for estimating the variance in this estimate. This estimate and variance *x* then formed the basis for a Gaussian probability distribution of the expected electron density at this point derived from NCS.

Several modifications to the simple model resulting in (4) were made to take into account local variations in similarity among NCS-related molecules and to reduce bias in the estimation of 〈*x* ^2^〉 arising from the iterative nature of density modification. The estimates of 〈*x* ^2^〉, the mean-square electron density shared by all NCS copies, are calculated in (3) as an average over the NCS asymmetric unit. In this formulation, all NCS copies and all points within the NCS asymmetric unit have the same value of 〈*x* ^2^〉. To take into account variation in the overall similarity between pairs of NCS-related molecules, an estimate *c*_*ij*_ = 〈*x* ^2^〉_*ij*_ was estimated separately for each pair. Then, to take into account local variations in similarity among NCS-related molecules, a local estimate of 〈*x* ^2^〉 corresponding to the local mean-square density in common between all NCS-related copies was obtained by using a locally averaged value of *c*(*x*) = 〈ρ_*i*_ρ_*j*_〉 in (3), where the local average was taken over a sphere with the same radius *r* used above in the identification of the NCS asymmetric unit. Then, the overall estimate of 〈*x* ^2^〉 for a particular pair of NCS-related points in molecules *i* and *j* was the product of *c*_*ij*_ and *c*(*x*).

In this density-modification procedure, the electron density at points in *n*_NCS_ − 1 NCS-related copies of the NCS asymmetric unit are use to estimate the expected density at points in the remaining copy. After one or more cycles of density modification, the density at all *n*_NCS_ copies of the NCS asymmetric unit tend to become increasingly similar. This is desirable of course, as the point of using NCS in density modification is to take advantage of the fact that the density in the various NCS copies really is more similar than is found in the initial map. However, in practice the density-modification procedure can sometimes make the NCS copies even more similar than they really are, leading to an overestimate of 〈*x* ^2^〉 and an underestimate of the variance 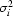. To obtain a less biased estimate of the variance 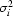, a cross-validation approach was used. At the start of the density-modification procedure several cycles were carried out without including NCS information in the phasing process, but including the calculation of 〈*x* ^2^〉. This overall covariance estimate was used as a ‘free’ or unbiased estimate throughout the density-modification procedure. Then, to account for local variation in the covariance of density 〈*x* ^2^〉, the overall values were multiplied for each cycle of density modification by the current ratio of the locally averaged value of 〈ρ_*i*_ρ_*j*_〉 (as described above) to the overall mean value of 〈ρ_*i*_ρ_*j*_〉.

### 2.4. Combination of information from NCS with *a priori* probability distribution of electron density in the macromolecule region

Even in the absence of NCS, a substantial amount of information exists on the expected distribution of electron density at points in the region of the macromolecule. As discussed earlier (Terwilliger, 2000 ▶), the *a priori* probability distribution of electron density in this region can be expressed in terms of the distribution found for model electron density, ρ_*M*_, 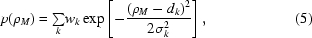
where the coefficients *w*_*k*_, *d*_*k*_ and 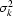 are estimated by fitting *p*(ρ_*M*_) to the model electron density. A similar description can be obtained for the solvent-containing region of the model map. The *a priori* probability distribution for electron density in the macromolecule-containing region of an experimental map with errors can then be described by
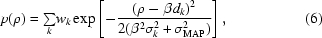
where the coefficients β and 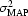 are estimated by fitting (6) and the corresponding equation for the solvent region (with the same values of β and 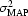 and different values of *w*_*k*_, *c*_*k*_ and 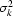) to the electron density in the experimental map. For simplicity we rewrite (6) as
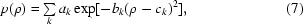
where
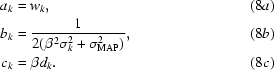
The NCS-based information consists of an estimate, ρ′, of the density at each point in the map, with an associated variance σ^2^,
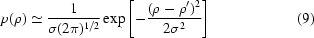
or
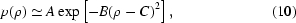
where,
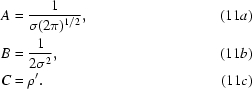
Combining this with (7) yields the expression
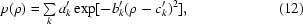
where the coefficients 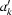, 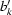 and 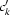 are given by
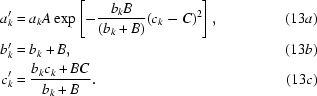

            

## 3. Results and discussion

### 3.1. Automatic identification and verification of NCS

The procedure for identifying (Terwilliger, 2002 ▶) and testing for NCS described here was tested by applying it to phases obtained from four MAD experiments and one SAD experiment on crystals with twofold, threefold, fourfold and sixfold NCS. In each case, the *SOLVE* software (Terwilliger & Berendzen, 1999 ▶) was used to identify selenium sites and calculate a starting electron-density map. The MAD data sets included a nucleotide diphosphate kinase with nine selenium sites from *Pyrobaculum aerophilum* (Pédelacq *et al.*, 2002 ▶), a hypothetical protein with 16 selenium sites from *P. aerophilum* (J. D. Pedelacq, E. Liong & T. C. Terwilliger, unpublished work), a red fluorescent protein with 26 selenium sites (Yarbrough *et al.*, 2001 ▶) and a formate dehydrogenase with 12 selenium sites from *P. aerophilum* (T. S. Peat, J. M. Newman, G. S. Waldo & T. C. Terwilliger, unpublished work; PDB entry 1qp8). The SAD data set was 2-aminoethylphosphonate transaminase with 66 selenium sites (Chen *et al.*, 2000 ▶).

Table 1 ▶ lists these crystals, with the number of NCS copies in the asymmetric unit, the number of NCS operators found from the selenium sites by the automatic procedure we developed recently (Terwilliger, 2002 ▶) and the number of NCS operators found after comparing the electron density at the potentially NCS-related positions. In all five cases, the NCS could be identified correctly from the heavy-atom sites and confirmed using the electron density in the map. In one of the cases (the dehydrogenase from *P. aerophilum)*, the NCS in the selenium sites could only be detected when the allowed deviation of the sites from perfect NCS was increased from the default value of 1.4 Å (half the resolution) to 3.0 Å.

As a further test, the procedure for identifying and verifying NCS was applied to three MAD data sets and one MIR data set in which the crystals had no NCS (Table 1 ▶). The MAD data sets were gene 5 protein (Skinner *et al.*, 1994 ▶), the armadillo repeat region from β-catenin (Huber *et al.*, 1997 ▶) and initiation factor 5A from *P. aerophilum* (Peat *et al.*, 1998 ▶), and the MIR data set was dehalogenase from *Rhodococcus* (Newman *et al.*, 1999 ▶). In each case, the two-step procedure of searching for NCS in the heavy-atom sites and verifying any NCS that was present in the heavy-atom sites resulted correctly in the conclusion that no NCS was present. In two cases (β-catenin and dehalogenase), the correct conclusion was drawn after NCS was found in the heavy-atom sites but not in the electron-density map.

### 3.2. Incorporation of NCS in statistical density modification

Table 2 ▶ illustrates the utility of NCS information in the context of statistical density modification, comparing the quality of the final electron-density maps with and without the inclusion of NCS information. The quality of the maps was assessed using two measures. One was the mean effective figure of merit of the phases, calculated from the mean cosine of the phase difference between the phases from this procedure and the phases from a refined model. The other was the correlation coefficient of the map calculated from this procedure with the map calculated using phases from the refined model.

The extent of phase improvement resulting from the use of the NCS information varied considerably from case to case (Table 2 ▶). The starting correlation of the map obtained from MAD or SAD phasing with the map calculated from the refined models and the starting mean cosine of the phase error is listed for each. Additionally, the corresponding values for the maps obtained after statistical density modification, with and without NCS, are shown. In both of the cases with twofold symmetry, the NCS information improved the phasing very slightly. This was a little surprising considering that the twofolds were not parallel to crystallographic symmetry axes in either case and the weights on NCS were sufficient to bring the correlation coefficients between NCS copies to 0.81 (formate dehydrogenase) and 0.93 (the hypothetical protein).

In the case of threefold symmetry, the inclusion of NCS made a very large difference, increasing the correlation of the resulting map with the map calculated from the refined model from 0.65 to 0.77. Somewhat surprisingly, in the cases with four and six NCS-related molecules the inclusion of NCS had a relatively small effect. On the other hand, the phases were very accurate even without NCS (correlations with the model map of 0.90 and 0.79, respectively), so in these cases there might simply not be much additional information available from the inclusion of NCS. This conclusion was tested by creating a test set of phases from the red fluorescent protein data set using just four of the 26 sites for phasing. As anticipated, beginning with a map correlation of 0.29, the inclusion of NCS had a very large effect, raising the final map correlation from 0.33 to 0.85, nearly as high as that obtained using all the selenium sites for phasing.

### 3.3. Estimation of variances in estimates of electron density from NCS

A key element of the statistical method for density modification is the ability to specify a probability distribution for the expected electron density in a map. In the case of NCS, this means that the method can, in principle, take into account the similarity of NCS-related molecules. It can also take into account the differences between NCS-related copies in a map that arise from errors in phases. In the present implementation of NCS in statistical density modification, (4) is used to estimate the uncertainty in target values of electron density used as prior information based on NCS. We tested whether these estimates are optimal by carrying out a systematic investigation of the phase improvement obtained when these variance estimates are multiplied by each of a range of values from 0.1 to 150.

**Figure 1:**
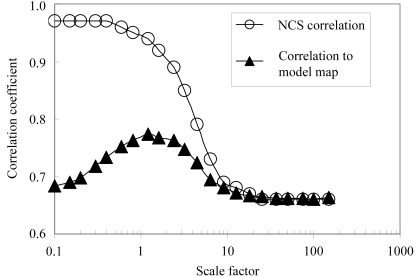
Effect of scale factor multiplying NCS variance estimates on correlation to model map and on NCS correlation.

Fig. 1 ▶ shows the result of this test applied to the NDP-kinase data, with the threefold NCS applied. When NCS variance estimates are multiplied by a large scale factor (150) so that NCS is effectively not used in density modification, the final correlation of the NCS-related regions of the map after density modification was just 0.66 and the correlation between the density-modified map and the map based on the refined structure of NDP-kinase was 0.66 (Fig. 1 ▶). At the other extreme, when variance estimates are multiplied by a small scale factor (0.1) so that NCS is strongly emphasized in density modification, the final correlation of the NCS-related regions of the map was 0.97, but a map correlation between the density-modified map and the map based on the refined structure of NDP-kinase is still only 0.68. At intermediate values of the scale factor (*e.g.* 0.8–1.2), corresponding closely to the estimates of the variance obtained with (4), NCS is included in density modification, but in a balanced way. The resulting final correlation of the NCS-related regions of the map is 0.94–0.95 and the final map correlation with the model map is 0.76–0.77. Overall, Fig. 1 ▶ indicates that the estimates of variances obtained using (4) are very close to optimal in carrying out statistical density modification with NCS.

## 4. Conclusions

The map-probability function in statistical density modification provides a way to incorporate information using a different probability distribution of expected values of electron density for every point in a map. This flexibility means that it is not necessary to assume that all NCS-related copies of a molecule in a crystal are identical, or even to assume that all parts of a single molecule are equally similar to the NCS-related parts of another molecule. The extent of local similarity among NCS-related molecules can be assessed using the local correlation of density and bias in these estimates owing to the iterative nature of density modification can be reduced by estimating the overall correlation by cross-validation.

The methods described here are implemented in the software *RESOLVE* (Terwilliger, 2000 ▶) available from http://solve.lanl.gov.

## Figures and Tables

**Table 1:** Automatic identification and evaluation of NCS from heavy-atom sites

Structure	No. of sites found by *SOLVE*	NCS (actual)	NCS (found from heavy-atom sites)	NCS (found in electron-density map)
NDP kinase	9	Threefold	Threefold	Threefold
Hypothetical	16	Twofold	Twofold	Twofold
Red fluorescent protein	26	4 copies	4 copies	4 copies
2-Aminoethylphosphonate (AEP) transaminase	66	6 copies	6 copies	6 copies
Formate dehydrogenase	12	Twofold	Twofold†	Twofold
Gene 5 protein	2	None	None	None
Armadillo repeat from β-catenin	15	None	2 copies	None
Dehalogenase	13 (unique sites in 5 derivatives, plus 5 duplicate sites not included)	None	3 copies	None
Initiation factor 5A	4	None	None	None

† Only identified automatically when tolerance for NCS in heavy-atom sites was increased from 1.4 to 3 Å.

**Table 2:** Use of NCS in statistical density modification

	Map correlation			〈cos(Δϕ)〉		
		Density-modified			Density-modified	
Structure	*SOLVE*	No NCS	NCS	*SOLVE*	No NCS	NCS
NDP kinase	0.40	0.65	0.77	0.27	0.42	0.54
Hypothetical	0.42	0.58	0.62	0.50	0.61	0.68
Red fluorescent protein	0.76	0.90	0.91	0.70	0.79	0.80
Red fluorescent protein (using only 4 of 26 Se sites from *SOLVE*)	0.29	0.33	0.85	0.23	0.26	0.66
2-Aminoethylphosphonate (AEP) transaminase	0.64	0.79	0.81	0.52	0.63	0.66
Formate dehydrogenase	0.48	0.77	0.77	0.29	0.50	0.52
